# Effect of D222G Mutation in the Hemagglutinin Protein on Receptor Binding, Pathogenesis and Transmissibility of the 2009 Pandemic H1N1 Influenza Virus

**DOI:** 10.1371/journal.pone.0025091

**Published:** 2011-09-22

**Authors:** Jessica A. Belser, Akila Jayaraman, Rahul Raman, Claudia Pappas, Hui Zeng, Nancy J. Cox, Jacqueline M. Katz, Ram Sasisekharan, Terrence M. Tumpey

**Affiliations:** 1 Influenza Division, National Center for Immunization and Respiratory Diseases, Centers for Disease Control and Prevention, Atlanta, Georgia, United States of America; 2 Harvard-MIT Division of Health Sciences and Technology, Singapore-MIT Alliance for Research and Technology, Department of Biological Engineering, Koch Institute for Integrative Cancer Research, Massachusetts Institute of Technology, Cambridge, Massachusetts, United States of America; Hallym University, Republic of Korea

## Abstract

Influenza viruses isolated during the 2009 H1N1 pandemic generally lack known molecular determinants of virulence associated with previous pandemic and highly pathogenic avian influenza viruses. The frequency of the amino acid substitution D222G in the hemagglutinin (HA) of 2009 H1N1 viruses isolated from severe but not mild human cases represents the first molecular marker associated with enhanced disease. To assess the relative contribution of this substitution in virus pathogenesis, transmission, and tropism, we introduced D222G by reverse genetics in the wild-type HA of the 2009 H1N1 virus, A/California/04/09 (CA/04). A dose-dependent glycan array analysis with the D222G virus showed a modest reduction in the binding avidity to human-like (α2-6 sialylated glycan) receptors and an increase in the binding to avian-like (α2-3 sialylated glycan) receptors in comparison with wild-type virus. In the ferret pathogenesis model, the D222G mutant virus was found to be similar to wild-type CA/04 virus with respect to lethargy, weight loss and replication efficiency in the upper and lower respiratory tract. Moreover, based on viral detection, the respiratory droplet transmission properties of these two viruses were found to be similar. The D222G virus failed to productively infect mice inoculated by the ocular route, but exhibited greater viral replication and weight loss than wild-type CA/04 virus in mice inoculated by the intranasal route. In a more relevant human cell model, D222G virus replicated with delayed kinetics compared with wild-type virus but to higher titer in human bronchial epithelial cells. These findings suggest that although the D222G mutation does not influence virus transmission, it may be considered a molecular marker for enhanced replication in certain cell types.

## Introduction

The 2009 H1N1 pandemic influenza virus spread from its first detection in humans in March 2009 to all populated continents in a matter of weeks, causing over 18,000 laboratory-confirmed deaths reported to WHO in over 215 countries [1]. Although the WHO assessed the impact of the H1N1 pandemic as moderate since the majority of infected patients experienced mild influenza-like illness (www.who.int), the 2009 H1N1 virus caused a wide spectrum of influenza-related complications in children less than 5 years of age, pregnant women, and those with certain underlying comorbidities [2], [3], [4], [5]. Although preexisting immune status and underlying chronic medical conditions are recognized risk factors for severe disease, the viral molecular determinants governing virulence of the 2009 H1N1 virus are poorly understood.

Previous studies on highly pathogenic influenza viruses have contributed to the identification of numerous molecular markers associated with adaptation to human hosts and increased virulence and transmissibility, including key amino acids at the 627 and 701 positions in PB2 and the presence of full-length PB1-F2 and NS1 proteins [6], [7], [8], [9]. However, the analysis of 2009 H1N1 viruses revealed an absence of these known molecular determinants, underscoring the virus-specific nature of such determinants and the existence of as yet unrecognized molecular features that contributed to the establishment of the 2009 H1N1 virus as a pandemic strain with moderate virulence [10]. A potential virulence marker associated with 2009 H1N1 viruses was first identified among patients in Norway, with a change from aspartic acid to glycine at position 222 (D222G) in HA1 present in 18% of clinical specimens from patients with severe disease but in 0% of those from mild cases [11]. Subsequent reports from cases worldwide further strengthened this association [12], [13], [14], [15], [16], [17]. These retrospective analyses have found that cases bearing the D222G mutation were more likely to be associated with severe pneumonia, admission to intensive care facilities, and death [18]. Several studies in *in vitro* and *in vivo* models have examined the role of D222G in 2009 H1N1 virus virulence with varying outcomes. The majority of studies have reported that presence of D222G is sufficient to enhance virus replication and lethality in mouse models, with this effect ranging from modest to pronounced [19], [20], [21]. However, other groups have not observed substantial differences between wild-type and D222G viruses in mouse or ferret models [22], indicating the need for further investigation into the role of D222G in virulence of 2009 H1N1 pandemic viruses.

The location of position 222 in the receptor binding site of HA predicts that alteration of this position would influence the binding specificity of a virus bearing this mutation [23]. Recent studies have reported that the D222G mutation confers enhanced binding to α2-3 linked sialic acids, suggesting a greater ability to bind to lung cells in the lower respiratory tract in humans and cause an exacerbation of disease [22], [24]. In the present study, a 2009 H1N1 virus A/California/04/09 (CA/04) was mutated to generate the HA D222G mutation in order to study the role of D222G in pathogenesis and transmission in both ferret and mouse models. A dose-dependent direct binding glycan array analysis of virus carrying the D222G HA mutation showed observable changes in binding to glycans terminated by both α2-6 and α2-3-linked sialic acids. Although the presence of this mutation did not cause a significant increase in pathogenesis and transmission in ferrets, it caused heightened virulence of this virus in mice and exhibited enhanced replication in human respiratory cells in comparison to the wild-type CA/04 virus.

## Results

### Effect of D222G mutation on the receptor binding properties of HA

Asp222 in the wild-type CA/04 HA is a characteristic residue predominantly found in human-adapted H1N1 viruses. This residue has been shown to play a key role in providing optimal contacts with the penultimate Gal sugar in glycans terminated by α2-6-linked sialic acid [25], [26]. Therefore a D222G mutation is likely to affect the molecular contacts of this key residue position with the glycan receptor. It was demonstrated previously that an analogous mutation in the prototypic 1918 pandemic (A/South Carolina/1/1918 or SC18) HA resulted in shifting its glycan receptor-binding from high specificity for α2-6 sialylated glycans to a mixed binding to both α2-6 (albeit at a lower affinity than wild-type HA) and α2-3 sialylated glycans [27], [28].

**Figure 1:**
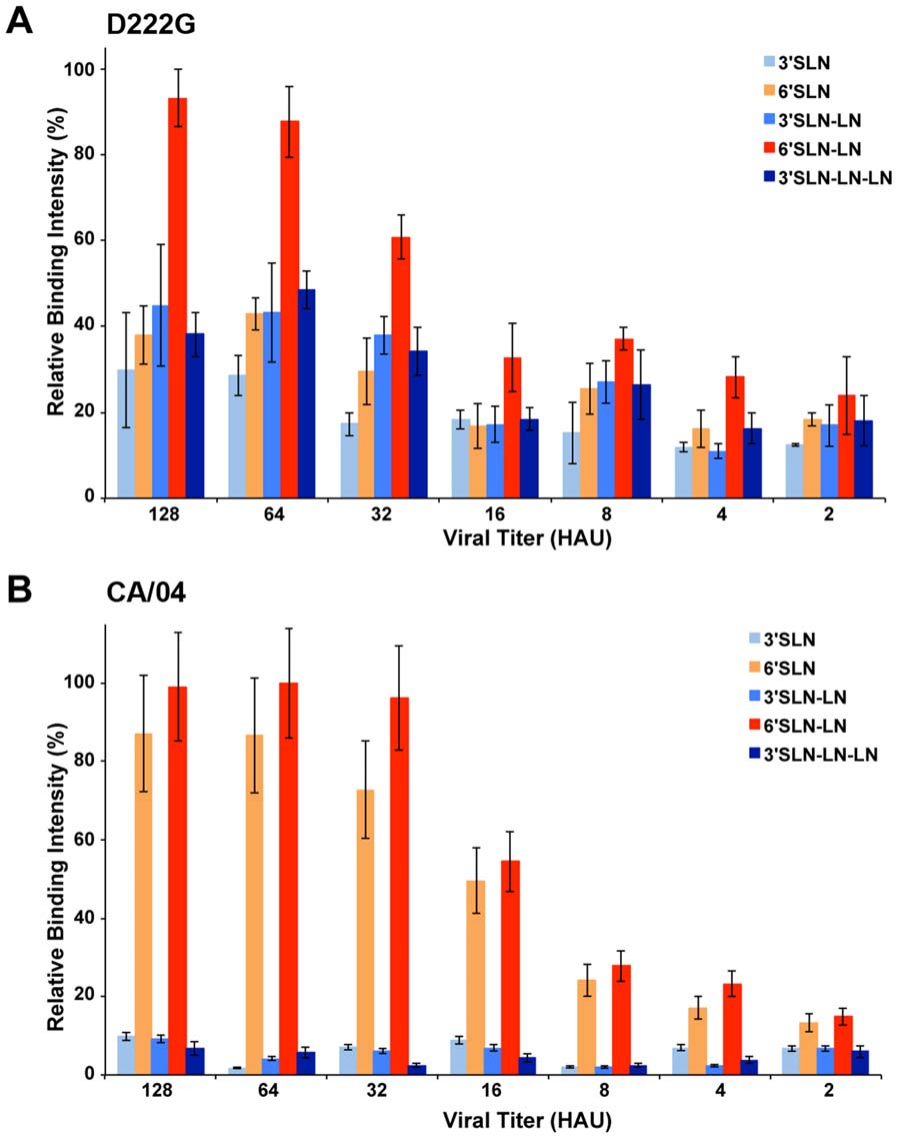
Dose-dependent glycan array-binding of CA/04 D222G virus. Dose-dependent binding of CA/04 D222G (A) or wild-type CA/04 (B) virus to representative α2-3 and α2-6 sialylated glycans on the glycan array. The y-axis shows percentage of maximum binding signal intensities. In both cases saturation binding signals were observed at viral titers of 128 HAUs. The chemical description of glycans is provided in Table S1. The 6′SLN-LN glycan is representative of the predominant glycan motifs expressed in the human upper respiratory tract.

To investigate the effect of D222G mutation on glycan-receptor binding properties of CA/04 virus, we analyzed the binding of both wild-type and mutant virus in a dose-dependent fashion to representative α2-3 and α2-6 sialylated glycans on a glycan array platform (Figure 1). Our analysis showed that D222G mutation resulted in a modest reduction in the binding avidity to α2-6 sialylated glycans (6′SLN and, to a lesser extent, 6′SLN-LN) in comparison with the wild-type virus. The notable difference in glycan-binding properties was the substantial increase in binding of D222G mutant virus to α2-3 sialylated glycans (relative to its α2-6 binding) compared with that of wild-type virus. These results with CA/04 virus are consistent with previous observations demonstrating an increase in α2-3 binding of other 2009 H1N1 viruses carrying the D222G mutation [24].

### Effect of D222G mutation on pathogenesis and respiratory droplet transmission

A recent study demonstrated that the introduction of D222G in a 2009 H1N1 virus did not alter the pathogenicity or transmissibility of a 2009 H1N1 isolate A/Netherlands/602/09 in ferrets [22]. However, the use of a parental strain which already possesses a highly transmissible phenotype may limit the ability to identify additional molecular changes which could confer enhanced transmissibility [29]. In contrast, the 2009 H1N1 CA/04 virus, which exhibits reduced transmissibility in the ferret model compared with seasonal H1N1 viruses, and previously enabled us to identify an HA mutation which conferred efficient transmission by respiratory droplets in ferrets was used in this study to more stringently assess the impact of D222G on virus transmission [30], [31].

Ferrets inoculated with the D222G virus exhibited similar clinical signs as CA/04 wild-type virus and a reverse-genetics derived CA/04 virus, with ferrets exhibiting transient weight loss (mean maximum weight loss of 11.3% on day 6–7 p.i.) and fever (mean maximum fever 1.9°C over baseline day 2 p.i.) before returning to baseline levels [30], [31]. D222G virus-infected ferrets further exhibited a mild, transient lymphopenia (22% decrease in circulating lymphocytes on day 3 p.i.) which was comparable to ferrets infected with other 2009 H1N1 viruses [32]. D222G virus was detected in the ferret upper respiratory tract (6.7±0.2 log_10_ PFU/ml nasal turbinates) and the lower respiratory tract (6.3±0.6 log_10_ PFU/g lung tissue) at similar titers as 2009 H1N1 wild-type viruses day 3 p.i. [22]. Moreover, the D222G mutant virus was detected in the intestinal tract of 1/3 ferrets day 3 p.i. (2.29 log_10_ PFU/ml), and in rectal swabs collected on days 1 and 3 p.i. in 2/3 ferrets (average titer 1.4±0.2 log_10_ PFU/ml), comparable to other 2009 H1N1 isolates [31].

**Figure 2:**
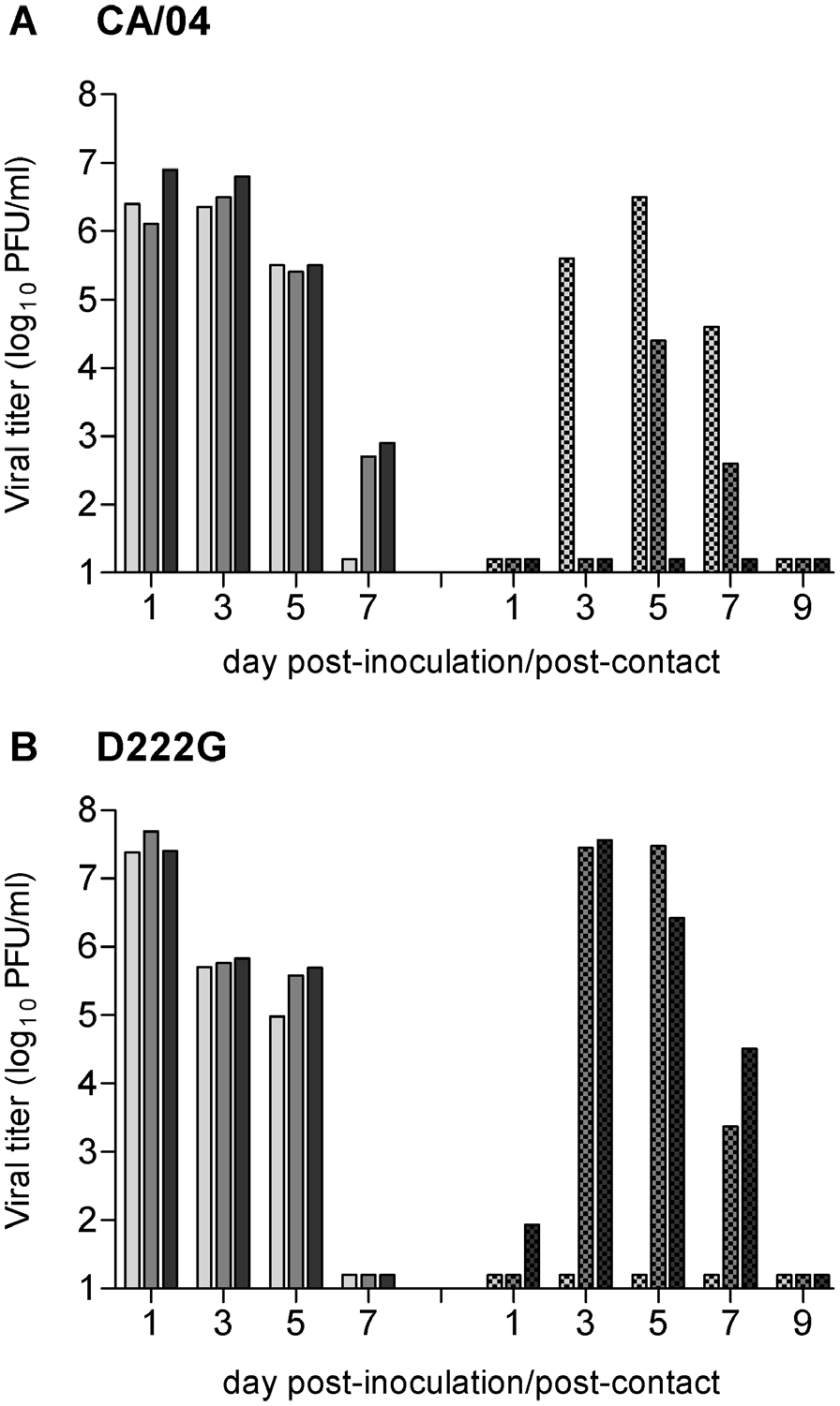
Respiratory droplet transmissibility of D222G virus. Three ferrets were inoculated with 10^6^ PFU of reverse-genetics CA/04 (published previously in [30]) (A) or D222G (B) virus. All ferrets were housed individually in specialized cages that permit exchange of respiratory droplets, but prevent direct or indirect contact between inoculated-contact animal pairs. A naïve ferret was placed in an adjacent cage to an inoculated ferret 24 hrs p.i. Viral replication was determined by titration of nasal washes collected on alternate days from inoculated (left bars) and contact (right bars) ferrets. The limit of virus detection was 10 PFU/ml.

To evaluate the transmissibility of D222G virus by respiratory droplets, three ferrets were inoculated with 10^6^ PFU of virus. Approximately 24 hours p.i., inoculated-contact pairs were established by placing naïve ferrets in each of three adjacent modified cages with a perforated side wall, allowing air exchange between ferrets in the absence of direct or indirect contact. Nasal washes (NW) were collected on alternate days p.i. or post-contact (p.c.) for virus titration. Detection of infectious virus in NW of contact ferrets and seroconversion of contact ferrets were considered evidence of virus transmission. Consistent with the experimental transmission data obtained with wild type 2009 H1N1 viruses [31], CA/04 virus did not spread by respiratory droplet to every contact ferret; infectious virus was detected in the nasal washes of two of three contact ferrets (Figure 2A) [30]. D222G virus replicated efficiently in the upper respiratory tract of inoculated ferrets, reaching peak mean titers of 7.5±0.2 log_10_ PFU/ml day 1 p.i., significantly higher than CA/04 virus at this time (p<0.03) [30]. Similar to CA/04 virus, D222G mutant virus was detected in NW of two of three contact ferrets, with virus titers >7 log_10_ PFU/ml on day 3 p.c. (Figure 2B). While detectable virus was not observed in NW of the third contact ferret, all three contact ferrets seroconverted to homologous virus by day 20 p.i. (HI titer range 320–640). This pattern of D222G virus transmission by respiratory droplets was confirmed in a duplicate experiment that resulted in virus detection in NW in two of three contact ferrets and seroconversion of all contact ferrets (data not shown). Ferret lung and NW samples from inoculated and contact animals that were harvested were sequenced to confirm the mutation. All samples yielded the expected mutant sequence; reversion of the mutation (222G to 222D) was not observed.

### Enhanced virulence of D222G virus in mice by the intranasal but not intraocular route

To determine whether the D222G mutation conferred heightened virulence of a 2009 H1N1 virus in a mouse model, we inoculated mice i.n. with 10^5^ PFU of either CA/04 or D222G virus. Mice inoculated with D222G virus by the intranasal route exhibited greater weight loss than CA/04 virus-inoculated mice; one mouse from the D222G group was euthanatized on day 11 p.i. due to excessive weight loss (Table 1). However, neither virus exhibited >50% lethality in mice when inoculated at this dose. While both viruses replicated efficiently in the lungs of mice on days 3 and 6 p.i., D222G virus replicated to a significantly higher mean titer compared with CA/04 virus at day 6 p.i. (p<0.05). Virus was found infrequently and at low titer in the nose and intestines of mice from both groups on day 3 p.i. only (Table 1 and data not shown). Similar titers of virus in the lung and sporadic detection of virus in intestinal tissue with these viruses was observed with numerous other 2009 H1N1 viruses in this model [33].

**Table 1:** Pathogenesis of CA/04 and 222G viruses following intranasal inoculation in mice.

			Viral titer^b^			
Virus	% wt loss^a^	Survival	Lung (D3)	Nose (D3)	Intestine (D3)	Lung (D6)
CA/04	15.8% (4)	5/5	6.0±0.3 (3/3)	2.1 (1/3)	1.5 (1/3)	5.1±0.5 (2/3)
D222G	22.1% (10)	4/5	6.2±0.1 (3/3)	1.7±0.6 (2/3)	1.7 (1/3)	5.9±0.1 (3/3)^c^

*^a^*Mean maximum percent weight loss (5 mice per group) following inoculation with 10^5^ PFU. Day post-inoculation (p.i.) indicated in parentheses.

*^b^*Virus endpoint titers are expressed as the mean log_10_ PFU/ml plus standard deviation. Day 3 (D3) and Day 6 (D6) p.i. are shown. Numbers of mice with detectable virus included in the mean are indicated in parentheses.

*^c^*p<0.05 compared with CA/04 virus by Student's t test.

A recent report indicated that the presence of D222G enhanced the ability of a 2009 H1N1 virus to cause ocular disease in mice, with virus detected in the eye of one mouse following intranasal inoculation [22]. We did not observe extrapulmonary spread of virus to the eyes of mice following intranasal inoculation with either CA/04 or D222G virus, and ocular disease was not observed during visual examination of mice during the course of infection (data not shown). However, to better assess the ability of the D222G mutation to cause ocular disease in mice, we inoculated mice by the ocular route with both viruses. The right eye of each mouse was lightly scarified with a 2-mm corneal trephine blade, and 10^5^ PFU of each virus in a 5 µl volume was deposited onto the corneal epithelial surface and massaged into the eye with the eyelid. Mice inoculated by the ocular route with either virus did not exhibit substantial morbidity, and virus was not detected in the eye, nose, intestine, or lung on day 3 or 6 p.i. (data not shown). These results indicate that CA/04 virus is not well suited to infect mice by the ocular route, and the presence of D222G does not confer an ocular tropism in this model.

### D222G mutation does not alter 2009 H1N1 virus tropism in human respiratory and ocular cells

**Figure 3:**
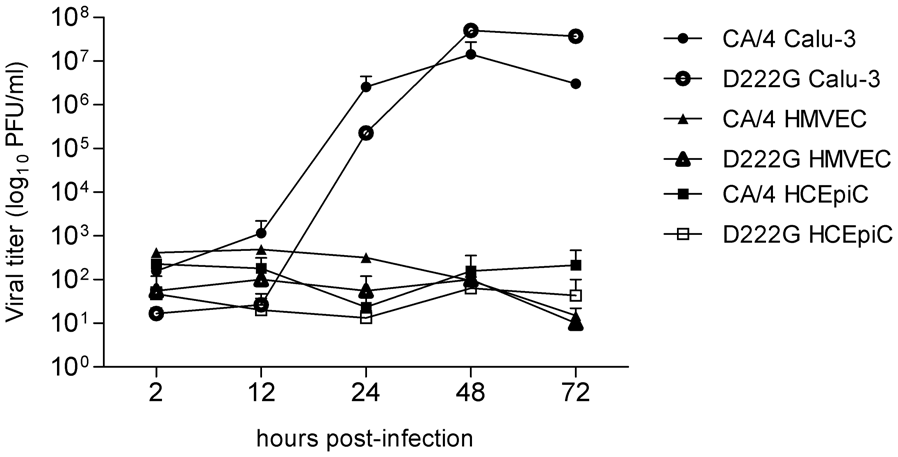
Replication kinetics of CA/04 and D222G viruses in human ocular and respiratory cells. Calu-3 (circles), HMVEC-LBI (triangles), or HCEpiC (squares) cells were infected at a MOI of 0.01 with the indicated viruses (CA/4, closed symbols; D222G, open symbols). Culture supernatants were removed at indicated times p.i., and titers were determined for infectious virus by standard plaque assay. The limit of virus detection was 10 PFU/ml. The mean from triplicate independent cultures per virus ± standard deviation is shown.

Previous studies have demonstrated that select influenza viruses which do not demonstrate an ocular tropism in mice nonetheless are capable of high-titer replication in human ocular cells [34]. To rule out the possibility that the D222G mutation confers an ocular tropism not observed in our murine model, we compared the ability of CA/04 and D222G viruses to replicate in primary human corneal epithelial cells (HCEpiC), which predominantly express α2-3 linked sialic acids [34]. HCEpiC were infected at a MOI of 0.01 and supernatants were collected at indicated times p.i. to quantify infectious virus (Figure 3, open symbols). Both CA/04 and D222G viruses were detected at low levels (<3 log_10_ PFU/ml) through 72 hrs p.i. in this cell type. In contrast, both viruses replicated efficiently in the human bronchial epithelial cell line Calu-3 (Figure 3, closed symbols). Similar to a previous study, we observed a slight delay in D222G virus replication in Calu-3 cells at 24 hrs p.i., with titers of D222G virus being 10-fold lower than CA/04 virus (p<0.02) [22]. However, at 48 and 72 hrs p.i., D222G virus replicated to significantly higher titer than CA/04 virus in the human respiratory cells (p<0.0005). Primary human lung blood microvascular endothelial cells (HMVEC-LBI) express a greater proportion of α2-3 linked sialic acids compared with Calu-3 cells (H. Zeng, personal communication), but did not support efficient replication of either CA/04 or D222G viruses. Taken together, these findings indicate that the D222G mutation confers enhanced replication in respiratory epithelial cells but does not alter the ocular tropism of 2009 H1N1 virus.

## Discussion

The detection of D222G among severe and fatal cases of human infection has resulted in analyses to determine if this mutation represents the first virulence marker associated with 2009 H1N1 pandemic viruses. Previous studies have investigated the effect of D222G HA mutation on glycan-binding using binding assays such as red blood cell agglutination and binding to glycan arrays at saturating viral titers [22], [24]. While these assays serve as good screening tools to understand the extent of α2-3 or α2-6 binding, they were not designed to understand relative binding-avidity of the virus in a dose-dependent fashion. In this study, we used a dose-dependent direct binding assay, mammalian models, and human *in vitro* cultures to evaluate the relative contribution of this mutation to the virulence of a 2009 H1N1 virus. We demonstrate that a single D222G amino acid change augments binding to α2-3 linked sialic acids and results in higher overall titers of infectious virus in human airway cells and mouse lungs compared with wild-type virus. However, this change is not sufficient to enhance disease or confer efficient transmission of this virus by respiratory droplets in the ferret model and does not augment the ability to replicate in α2-3-rich human or murine ocular tissue.

The consequence of the D222G mutation on viral replication kinetics has been assessed previously in several relevant cell lines. Compared with wild-type virus, a D222G-bearing virus replicated to higher titers in human alveolar basal epithelial A549 cells, although generally similar kinetics between D222G and wild-type viruses were observed in the human placental cell line JEG-3 and MDCK cells [20], [21], [22]. We observed lower viral titers of D222G virus compared with CA/04 virus 24 hrs p.i. in Calu-3 cells, in agreement with a previous study using MDCK cells which also noted reduced viral titers with a virus bearing this mutation at this time p.i. [22]; however, significantly higher titers of D222G virus compared with wild-type virus were detected in Calu-3 cells at 48 and 72 hrs p.i. Interestingly, similar viral replication kinetics were observed in our murine model, with comparable titers between CA/04 and D222G viruses in the lungs of mice on day 3 p.i. but higher D222G virus titers in this tissue at day 6 p.i. However, the enhanced ability of D222G virus to bind to α2-3 linked sialic acids did not confer the ability to replicate efficiently in respiratory endothelial cells. Along with these previously published studies, our data suggests that the presence of D222G can enhance viral replication in some cell types, but this contribution is modest and is only apparent at later stages of infection.

Previous studies have demonstrated that, in general, 2009 H1N1 viruses are not highly pathogenic in mice [33], [35]. However, these viruses are capable of acquiring a lethal phenotype in mice following acquisition of key mutations, one of which is D222G [20], [36]. A previous study found that the D222G mutation on the CA/04 background conferred greater morbidity, mortality, and heightened virus replication in the lungs of mice compared with a wild-type virus, which is in agreement with our results [19]. An additional study performed a similar assessment of virulence with this mutation, but the use of a parental strain which is lethal for mice may have masked subtle differences conferred by the D222G mutation [22]. In the ferret model, the D222G mutation on the CA/04 background did not confer enhanced virulence, in accordance with previous results [22]. Thus, results in the ferret model have not supported a role of D222G in enhancing 2009 H1N1 virus virulence. Extrapulmonary spread of virus to the intestinal tract was detected following infection with both CA/04 and D222G viruses, a feature shared by numerous wild-type 2009 H1N1 viruses [31]. However, D222G virus failed to spread to other tissues following infection in either mice or ferrets. The enhanced replication and morbidity in mice, but not ferrets following D222G virus infection, is consistent with augmented binding to α2-3 sialylated glycans and the known predominance of α2-3 sialic acids in the mouse model [37].

Unlike seasonal influenza viruses which exhibit efficient transmission by respiratory droplets in the ferret model, our studies have shown that most 2009 H1N1 viruses and triple-reassortant swine H1N1 viruses possess reduced transmissibility by this route [29], [31], [32], [38]. The 2009 H1N1 viruses consistently transmitted to two of three ferrets, as measured by virus detection in nasal washes (Figure 2A and [31]). Although a single base-pair change (leading to the I216K mutation) in CA/04 HA can lead to efficient transmission in ferrets [30], we found that the D222G mutation did not appear to significantly enhance or diminish transmission (4 of 6 naïve contact ferrets had detectable virus in nasal washes) of the 2009 H1N1 virus. The enhanced transmission observed with the HA (Ile219→Lys) may be due, at least in part, to its increased human receptor-(α2-6) binding affinity (particularly to the 6′SLN-LN glycan) by several-fold in comparison with wild-type [30]. However, differences in receptor binding specificity between CA/04 and D222G did not preclude efficient replication of both viruses in ferret nasal wash samples. The inability of D222G virus to confer efficient respiratory droplet transmission may be related to the findings that this particular HA mutation does not substantially improve α2-6 binding avidity of the virus. In fact, molecular epidemiologic studies revealed that the majority of 2009 H1N1 viruses circulating during the pandemic did not bear the D222G mutation, further suggesting that this mutation is not a determinant of virus transmissibility [11], [39].

While rare, reports of conjunctivitis following 2009 H1N1 virus infection have been documented [17], [40], [41]. Studies have also shown that select 2009 H1N1 viruses are capable of replicating in human ocular cell types, albeit to lower titer than avian influenza viruses [42]. However, it appears that 2009 H1N1 viruses, similar to seasonal influenza viruses, are not well-suited to use the eye as a portal of entry, as ocular infection of mice with these viruses fails to result in a productive virus infection [33]. In the present study, the presence of D222G did not confer the ability of this virus to infect mice by the ocular route or the ability to replicate more efficiently compared with wild-type CA/04 virus in human corneal epithelial cells. The detection of macroscopic ocular symptoms in mice infected with a virus bearing the D222G mutation in a previous study may be due to strain-specific differences between parental viruses used or laboratory-specific conditions, but does not appear to be a universal property of 2009 H1N1 viruses possessing this mutation [22]. Nonetheless, the lack of productive replication of D222G virus in human ocular cells indicates that the enhanced cell tropism present with this virus, observed in the ability to bind both ciliated and non-ciliated cells in HTBE cell cultures, does not translate to all cell types [24].

The relatively rapid emergence of D222G during serial passage in mice, cells, or eggs indicates the ability of 2009 H1N1 viruses to acquire this mutation under the right conditions, and the lack of reversion observed in this study suggests that this mutation is stably maintained. [20], [24], [36], [43]. In support of this, numerous studies have reported that the mutation arises during course of human influenza (2009 H1N1) infection [11], [12], [13]. Further study is needed to better understand other molecular markers of the 2009 H1N1 virus that are known to be associated with severe disease and the role of these mutations on the virulence of other virus subtypes. This work underscores the importance of studying the contribution of virulence markers such as D222G on parental strains that do not themselves possess heightened virulence to avoid masking subtle differences which, while minor, nonetheless result in a detectable increase in virus pathogenicity in mammals. These results taken together with those of others demonstrate that it can be difficult to unequivocally demonstrate experimentally that a particular mutation is associated with enhanced severity since different animal models yield different results. In addition, subtle differences in viruses, experimental conditions and other factors may result in somewhat different results from different laboratories; nevertheless, the association of the D222G mutation with more severe human infections highlights the need to continue research in this area.

## Materials and Methods

### Rescue of recombinant influenza A viruses

The eight reverse-genetics plasmids used for the rescue of recombinant influenza A/California/04/09 (CA/04) virus were constructed as described previously [30], [44], [45]. The mutation from Asp to Gly at HA position 222 was achieved by altering the position 222 codons from GAT(D) to GGT(G) using a Stratagene QuikChange II Site-Directed Mutagenesis kit (Agilent Technologies, Santa Clara, CA) per manufacturer's instructions on the CA/04 HA plasmid template.

CA/04 and 222G viruses were rescued as described previously [30]. Briefly, 293T cells were transfected with the eight CA/04 plasmids and co-cultured with MDCK cells 12 hrs post-transfection. Rescued viruses were isolated by plaque purification on MDCK cells. Coding sequences of plasmids and rescued viruses were confirmed by automated sequencing performed at the Influenza Sequence Activity, Influenza Division, Centers for Disease Control and Prevention. Experiments with CA/04 and 222G viruses were conducted under biosafety level 3 containment with enhancements, in accordance with guidelines of the WHO. (https://www.who.int/csr/resources/publications/swineflu/Laboratorybioriskmanagement.pdf).

### Dose dependent direct binding of CA/04 D222G virus by glycan array

A streptavidin plate array comprising representative biotinylated α2→3 and α2→6 sialylated glycans as described previously [28], [30] (Table S1) was used for the analysis. 3′SLN, 3′SLN-LN, 3′SLN-LN-LN are representative avian receptors. 6′SLN and 6′SLN-LN are representative human receptors. The biotinylated glycans were obtained from the Consortium of Functional Glycomics through their resource request program. Streptavidin-coated High Binding Capacity Pierce 384-well Microplates (Pierce, Rockford, IL) were loaded to the full capacity of each well by incubating the well with 50 µl of 2.4 µM of biotinylated glycans overnight at 4°C. Excess glycans were removed through extensive washing with PBS. The viruses were diluted based on hemagglutinating units (HAU, obtained using 0.5% turkey erythrocytes) with 1X PBS+1% BSA. 50 µl of the diluted virus was added to each of the glycan – coated wells and incubated overnight at 4°C. This was followed by three washes with 1X PBST (1X PBS+0.1% Tween-20) and three washes with 1X PBS. Each of the wells was blocked with 1X PBS+1% BSA for 2 h at 4°C. The wells were incubated with primary antibody (ferret anti – CA/04 antisera; 1∶500 diluted in 1X PBS+1% BSA) and incubated for 5 h at 4°C. This was followed by three washes with 1X PBST and three washes with 1X PBS. Finally the wells were incubated with the secondary antibody (goat anti – ferret HRP conjugated antibody from Abcam; 1∶500 diluted in 1X PBS+1% BSA). The wells were washed with 1X PBST and 1X PBS as before. The binding signals were determined based on the HRP activity using the Amplex Red Peroxidase Assay (Invitrogen) according to the manufacturer's instructions. The assays were done in triplicate and appropriate negative controls were included.

### Ferret pathogenesis and transmission experiments

Male Fitch ferrets (Triple F Farms, Sayre, PA), 8–10 months of age and serologically negative by hemagglutination inhibition (HI) assay for currently circulating influenza viruses, were used in this study. Ferrets were housed for the duration of the experiment in cages within a Duo-Flow Bioclean environmental enclosure (Lab Products, Seaford, DE). The pathogenesis of each virus was assessed following intranasal (i.n.) inoculation of 10^6^ PFU of virus in a 1 ml volume, a dose reported to consistently infect ferrets with human or avian influenza viruses, was determined as previously described [46], [47]. Ferret hematologic and blood chemistry analyses were performed as previously described [32]. Transmission of virus by respiratory droplets was performed as previously described [38]. Nasal washes were collected from ferrets every other day for at least 9 days post-inoculation (p.i.) or contact (p.c.) and titrated by standard plaque assay using MDCK cells. Post-exposure serum was collected from virus-inoculated (15–19 days p.i.) and contact (18–21 days p.c.) ferrets and tested for H1-specific antibodies against homologous virus by HI assay using 0.5% turkey erythrocytes [48].

### Mouse pathogenesis and ocular tropism experiments

Female BALB/c mice (Charles River Laboratories, Wilmington, MA), 8–12 weeks of age, were inoculated i.n. with 10^5^ PFU of CA/04 or D222G virus in a 50 µl volume as previously described [33]. Ocular infection of mice with CA/04 and D222G viruses was performed as previously described using 10^5^ PFU in a 5 µl volume [34]. Five mice per group were monitored daily for 14 days p.i. for morbidity, as measured by body weight, and mortality. Any mouse which lost >25% of its pre-inoculation body weight was euthanatized. Three mice per group were euthanatized on days 3 and 6 p.i., and eye, nose, intestine, and lung tissues were collected to determine replication and systemic spread of virus. Tissues were homogenized in 1 ml of cold PBS, and clarified homogenates were titrated by standard plaque assay. Statistical significance for these and all experiments was determined using Student's *t* test.

### *In vitro* replication kinetics

The bronchial epithelial cell line Calu-3 (ATCC, Manassas, VA) was cultured on membrane inserts and infected apically as previously described [49]. Primary human corneal epithelial cells (HCEpiC) were obtained from ScienCell (San Diego, CA) at passage 1 and were prepared for virus infection as described previously [34]. Primary human lung blood microvascular endothelial cells (HMVEC-LBI) were obtained from Lonza (Walkersville, MD) and cultured with Endothelial Cell Basal Medium-2 (Lonza) (Zeng et al, in preparation). Virus was added to Calu-3, HCEpiC, or HMVEC-LBI monolayers at a multiplicity of infection (MOI) of 0.01 for one hour before washing. Cell type specific serum-free media was added to all wells with the addition of 300 µg/L N-*p*-tosyl-L-phenylalanine chloromethyl ketone-treated (TPCK) trypsin (Sigma-Aldrich, St. Louis, MO) in cultures using primary human cells. Aliquots of culture supernatant taken post-infection (p.i.) from triplicate cultures were immediately frozen at −80°C until titrated for the presence of infectious virus by standard plaque assay.

### Ethics Statement

All animal research described in this study was specifically approved by CDC's Institutional Animal care and Use Committee (IACUC). The animal research was conducted under the guidance of CDC's IACUC and in an Association for Assessment and Accreditation of Laboratory Animal Care International-accredited facility.

## Supporting Information

Table S1 **Expanded nomenclature of glycans used in the glycan array.** *^a^* Neu5Ac: N-acetyl D-neuraminic acid; Gal: D-galatose; GlcNAc: N-acetyl D-glucosamine. α/β: anomeric configuration of the pyranose sugars. All the sugars are linked via a spacer to biotin (-Sp-LC-LC-Biotin as described in http://www.functionalglycomics.org/static/consortium/resources/resourcecored5.shtml).
(DOC)
