# Supplementary material for: Effect of D222G Mutation in the Hemagglutinin Protein on Receptor Binding, Pathogenesis and Transmissibility of the 2009 Pandemic H1N1 Influenza Virus
Source: PLoS One. 2011 Sep 22;6(9):e25091. doi: 10.1371/journal.pone.0025091 (PMC3178596; doi:10.1371/journal.pone.0025091)
Supplement: Table S1 — Expanded nomenclature of glycans used in the glycan array. a Neu5Ac: N-acetyl D-neuraminic acid; Gal: D-galatose; GlcNAc: N-acetyl D-glucosamine. α/β: anomeric configuration of the pyranose sugars. All the sugars are linked via a spacer to biotin (-Sp-LC-LC-Biotin as described in http://www.functionalglycomics.org/static/consortium/resources/resourcecored5.shtml). (DOC) [file pone.0025091.s001.doc]

**Supplementary Table 1. Expanded nomenclature of glycans used in the glycan array.**

| **Glycan** | **Expanded nomenclature** *^a^* |
| --- | --- |
| 3’SLN | Neu5Acα2-3Galβ1-4GlcNAcβ1- |
| 6’SLN | Neu5Acα2-6Galβ1-4GlcNAcβ1- |
| 3’SLN-LN | Neu5Acα2-3Galβ1-4GlcNAcβ1-3Galβ1-4GlcNAcβ1- |
| 6’SLN-LN | Neu5Acα2-6Galβ1-4GlcNAcβ1-3Galβ1-4GlcNAcβ1- |
| 3’SLN-LN-LN | Neu5Acα2-3Galβ1-4GlcNAcβ1-3Galβ1-4GlcNAcβ1-3Galβ1-4GlcNAcβ1- |

*^a^* Neu5Ac: N-acetyl D-neuraminic acid; Gal: D-galatose; GlcNAc: N-acetyl D-glucosamine. α / β: anomeric configuration of the pyranose sugars. All the sugars are linked via a spacer to biotin (-Sp-LC-LC-Biotin as described in <http://www.functionalglycomics.org/static/consortium/resources/resourcecored5.shtml>)
